# An Exergoeconomic Analysis of a Gas-Type Industrial Drying System of Black Tea

**DOI:** 10.3390/e24050655

**Published:** 2022-05-06

**Authors:** Zhiheng Zeng, Bin Li, Chongyang Han, Weibin Wu, Xiaoming Wang, Jian Xu, Zefeng Zheng, Baoqi Ma, Zhibiao Hu

**Affiliations:** 1 College of Engineering, South China Agricultural University, Guangzhou 510642, China; zengzhiheng@stu.scau.edu.cn (Z.Z.); 20202009003@stu.scau.edu.cn (C.H.); ebianwxm1234@stu.scau.edu.cn (X.W.); xujianaa@stu.scau.edu.cn (J.X.); scauzzf@stu.scau.edu.cn (Z.Z.); mabaoqi@stu.scau.edu.cn (B.M.); huzhibiao1998@stu.scau.edu.cn (Z.H.); 2 School of Intelligent Manufacturing Engineering, Chongqing University of Arts and Sciences, Chongqing 404100, China; 20210011@cqwu.edu.cn

**Keywords:** exergoeconomic, exergy, industrial drying, black tea, water

## Abstract

The performance evaluation and optimization of an energy conversion system design of an energy intensive drying system applied the method of combining exergy and economy is a theme of global concern. In this study, a gas-type industrial drying system of black tea with a capacity of 100 kg/h is used to investigate the exergetic and economic performance through the exergy and exergoeconomic methodology. The result shows that the drying rate of tea varies from the maximum value of 3.48 g_water_/g_dry matter_ h to the minimum 0.18 g_water_/g_dry matter_ h. The highest exergy destruction rate is found for the drying chamber (74.92 kW), followed by the combustion chamber (20.42 kW) in the initial drying system, and 51.83 kW and 21.15 kW in the redrying system. Similarly, the highest cost of the exergy destruction rate is found for the drying chamber (18.497 USD/h), followed by the combustion chamber (5.041 USD/h) in the initial drying system, and 12.796 USD/h and 5.222 USD/h in the redrying system. Furthermore, we analyzed the unit exergy rate consumed and the unit exergy cost of water removal in different drying sections of the drying system, and determined the optimal ordering of each component. These results mentioned above indicate that, whether from an energy or economic perspective, the component improvements should prioritize the drying chamber. Accordingly, minimizing exergy destruction and the cost of the exergy destruction rate can be considered as a strategy for improving the performance of energy and economy. Overall, the main results provide a more intuitive judgment for system improvement and optimization, and the exergy and exergoeconomic methodology can be commended as a method for agricultural product industrial drying from the perspective of exergoeconomics.

## 1. Introduction

Drying is a traditional, simple and economic method used to reduce moisture from high-moisture-content products (e.g., natural products or industrials) to a specific amount of moisture content, while extending the storage time and improving the quality of the products [1]. However, drying is a high-energy-consumption operation in industrial production or agricultural product processing operations. According to the relevant literature, the energy consumption of industrial drying accounts for about 15% of the national total energy consumption, and the energy consumption cost accounts for 60–70% of the total cost [2,3,4]. Therefore, improving the drying energy efficiency and reducing the economic cost in the drying industry significantly promotes the development of the national environment and economy.

In recent years, based on concepts that include energy, exergy and economics, researchers have been put forth to improve the craft and equipment for drying to improve the energy efficiency and sustainability of the entire drying system to cope with energy and environmental concerns [5,6]. Among the methods developed by the researchers, exergy-based analysis methods have great potential for development [7]. For an energy system, exergy is the maximum work of energy output when the system moves from an unstable state to a stable state [8]. Exergy is a unified scale that reflects the quality and quantity of energy in a thermodynamic system. Compared with the traditional energy analysis methods, the advantages of exergy analysis methods are not only used to reveal the irreversibility in the energy conversion process of thermodynamic systems, but also to evaluate the sustainability and environmental impact of production systems [9,10,11]. In the last two decades, exergy analysis is limited to analyzing an energy system from a single perspective of energy; Rosen and Dincer innovatively proposed a new interdisciplinary exergy analysis method combining energy, environment and sustainable development [12,13]. Based on the above concept of exergy, the relevant research on the analysis of agricultural product drying systems using the energy method is listed in following Table 1.

As mentioned above, the main purpose of the exergy analysis method was to improve the energy efficiency of the overall energy system, so it only revealed the reasons and sources of energy loss in each component of the energy system from the perspective of energy [19,20]. Although the exergy-based approach evaluates the energy system in terms of energy, it does not by itself provide comprehensive evaluation criteria because it does not consider the linkages of the energy system to the economy and the environment [21,22]. Since exergy analysis-based methods have certain limitations, in addition to the energy performance and quality evaluation, an economic and environment evaluation of the energy system for the specific material should also be conducted. In the past three decades, relevant researchers proposed and used relevant methods to combine exergy with economics and the environment to evaluate the energy system [23,24,25,26], and it is widely used in energy-intensive systems, such as drying [27,28]. In general, the concept of thermodynamics combined with the concept of energy system economy and environment can provide solutions from multiple perspectives for energy system evaluation, optimization and energy conversion. Therefore, numerous works using the method of energy–exergy–economic analyses to optimize and improve energy conversion systems can be found in the literature [29,30,31].

From the viewpoint of energy–exergy and economics, the combination of thermodynamics and economics are increasingly popular engineering approaches for optimizing and improving the current agricultural product drying system at the component level. An advantage of this combination is the ingenious and unique combination of the thermodynamic and economic concepts of energy systems. For example, Ozturk M. et al. developed an integrated system consisting of a batch-type tea dryer and a PV/T unit, and used the exergoeconomic method to perform the analysis [32]. Singh A. et al. reported the effect of drying time on various energy, exergy, economic and exergoeconomic performance parameters in both a simple heat pump dryer (HPD) and SAHPD modes for the closed-system drying of banana chips [33]. Even though a number researchers used the exergoeconomic method to undertake the economic evaluation of the drying system, few works have reported on the application of the theory to industrial-scale Black tea drying systems [34,35,36]. In the present work, the existing advanced energy–exergy methodology is employed to estimate the energetic and exergetic performances of the existing gas-type industrial drying system of black tea, while an exergoeconomic methodology is adopted to reveal the costs related to each exergy stream and each component of the complex drying system. Furthermore, it is of great significance to analyze and optimize the energy consumption of each component by analyzing the damage rate of each component in the system, so as to improve the overall energy efficiency of the drying system of black tea.

## 2. Materials and Methods

### 2.1. Materials

**Figure 1:**
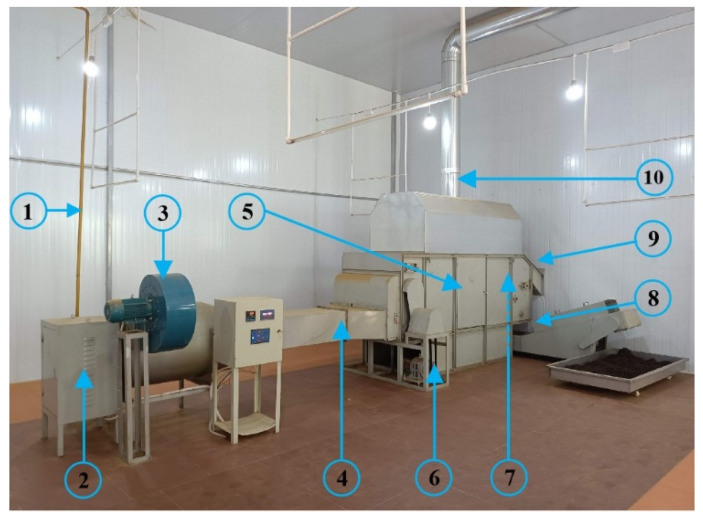
Picture of the black tea drying system: (1) gas pipeline; (2) gas control cabinet; (3) induced draft fan; (4) hot air inlet; (5) drying chamber; (6) chain plate motor; (7) hoist motor (behind the drying chamber); (8) dry tea outlet; (9) fermented tea inlet and (10) hot air outlet.

The tea leaves (tea variety: Yinghong NO.9) was freshly harvested from a local tea garden in Yangshan County, Guangdong Province. The average initial moisture content of the tea leaves after rolling and fermentation was 58.33% w.b., and the moisture content of dry tea after drying was 4.63% w.b.. The gas-type industrial drying system for black tea fermented leaves with a processing capacity of 100 kg/h is shown in Figure 1. The system operation parameters and service years are shown in Table 2.

### 2.2. Description and Working Principle of the Drying System

**Figure 2:**
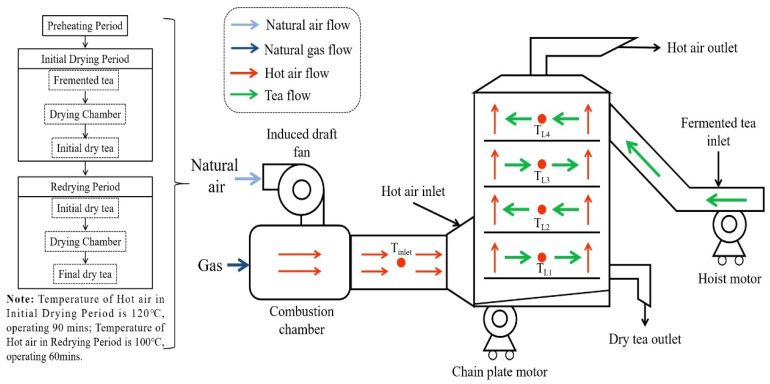
Schematic diagram of the drying system.

As can be clearly seen from Figure 2, the gas-type industrial drying system consists of five main components: combustion chamber (CC), induced draft fan (IDF), drying chamber (DC), hoist motor (HS) and chain plate motor (CPM). The overall drying operation of tea is mainly divided into three periods: **Preheating Period** (**PP**), **Initial Drying Period** (**IDP**) and **Redrying Period** (**RP**). **Preheating period**—Starting the machine, setting the initial inlet hot-air temperature to 120 °C, natural air heated by the gas burning was sent to the drying chamber by an induced draft fan to preheat the drying chamber for 3–5 min to stable temperature before tea drying. **Initial drying period**—The inlet temperature of the hot air for initial drying was maintained at 120 °C. The fresh fermented tea leaves were lifted by hoist motor to the top drying plate of the drying chamber from the feeding slot. The drying chamber consisted of four layers, and the adjacent two layers of the drying plates moved opposite to each other. The tea leaves were fed into the drying chamber from the top drying plate and driven from the feed side to the hot air inlet side by the moving chain plate. Therefore, on the hot air inlet side, the top layer of the tea leaves slid to the second layer and moved to the feed inlet side, then slid to the third layer, and so on. The movement of the tea leaves from the feeding inlet to the outlet needed a total of 12 min. **Redrying period**—Tea leaves dried in the initial drying period were flattened and naturally cooled to room temperature, and then redried. The inlet temperature of the hot air for redrying was setting to 100 °C. The speed of the chain plate motor was adjusted, differing from the initial drying state, and the movement of tea leaves in the redrying period from the feeding inlet to the outlet needed a total of 20 min. In addition, the weight of fermented tea in this experiment was about 180 kg, the initial drying time was 90 min and the redrying took 60 min; then, the final weight of the dry tea was approximately 45 kg. The schematic diagram of the drying operation system is shown in Figure 2. Details of the measurement instruments are shown in Table 3.

### 2.3. Data Record

During the overall drying operation, the temperatures of the hot air inlet (T_inlet_), four layers of the drying chamber (T_Li_, i = 1, 2, 3 and 4, especially the acquiescence T_L4_ = T_outlet_), was measured by thermal resistance sensors inserted into the corresponding components. The humidity of the inlet air flux (H_a,in_), outlet air flux (H_a,out_) and ambient air (H_0_) were measured by corresponding humidity sensors. The measured data were collected by a self-developed data acquisition system. Moreover, the moisture content of the drying tea in each layer of the drying chamber was calculated using the 105 °C constant weight methodology.

### 2.4. Drying Kinetics

In this experiment, the dry-basis moisture content, the wet-basis moisture content and the drying rate were adopted to analyze the drying kinetics of industrial black tea drying, which can be calculated according to the following Equations:

(1) $$MC_{wb}=\frac{m_{wet}-m_{d}}{m_{wet}}\times 100\%$$

(2) $$DR=\frac{MC_{\Delta t}}{\Delta t}$$

where *m_wet_* is the mass of wet tea, *m_d_* is the mass of dry tea, *MC* is the moisture content, *MC*_Δ*t*_ is the difference in moisture content per unit of time and Δ*t* is the unit of time.

### 2.5. Uncertainty Analysis

In the present work, the uncertainties of the obtained data were ascertained by means of the methodology introduced by Holman in 2001 [37]. The equation is shown in Equation (3). The results show that the uncertainties of the experimental data range from 0.4 to 2.8, indicating that the reliability of the data used for calculating the indicators adopted in the present work is good, in addition to confirming the reproducibility [38]:

(3) $$Z={\left[{\left(\frac{\partial y}{\partial x_{1}}x_{1}\right)}^{2}+{\left(\frac{\partial y}{\partial x_{2}}x_{2}\right)}^{2}+\ldots +{\left(\frac{\partial y}{\partial x_{n}}x_{n}\right)}^{2}\right]}^{1/2}$$

### 2.6. Theoretical Hypothesis

To investigate the drying system, several assumptions were taken into consideration in the present work, which are as follows:

1. The system was considered to be a stabilization system;
2. The ambient temperature and humidity were considered in a stable state;
3. The heat loss caused by heat conduction on the wall of the drying system was ignored;
4. The temperature and humidity gradient of the material in the drying process was ignored;
5. The energy loss caused by the differential pressure of the gas supply pipeline was ignored.

### 2.7. Energy–Exergy Analysis

The energy need for the drying system was supplied by natural gas, and the calculation of the combustion heat of natural gas is expressed as Equation (4) [39]:

(4) $$Q_{gas}=V_{gas}\times q_{gas}\;$$

where *q_gas_* is the calorific value per unit volume of natural gas, 3.56 × 10^7^ J/m^3^; *V_gas_* is the unit volume of natural gas.

The calculation of the heat required for natural air at normal temperature to rise to the setting temperature was performed by using Equation (5):

(5) $$Q_{a}=m_{g,a}c_{p,a}\left(T_{1}-T_{0}\right)$$

The energy loss of heating natural air was determined with Equation (6):

(6) $$Q_{loss}=Q_{gas}-Q_{a}$$

The efficient of heating natural air was calculated using Equation (7):

(7) $$\eta_{gas}=\frac{Q_{a}}{Q_{a}+W_{fan}}=\frac{m_{g,a}c_{p}\left(T-T_{0}\right)}{m_{g,gas}c_{p,a}\left(T_{1}-T_{0}\right)+P_{fan}t}$$

The equilibrium equation is generally used to analyze the stability of the exergy flow rate of the drying system, which is described in Equation (8) [40]:

(8) $$Ex_{sys,des}=Ex_{sys,in}-Ex_{sys,out}$$

where *Ex_sys,in_* is the inlet exergy of the drying system, *Ex_sys,out_* is the outlet exergy of the drying system and *Ex_sys,des_* is the exergy dissipation of the drying system.

We ignored the kinetic energy loss of hot air; the exergy inlet to the drying chamber was divided into two parts and calculated as Equation (9):

(9) $$Ex_{sys,in}=Ex_{fan}+Ex_{gas}$$

where the induced draft fan consuming mechanical exergy was calculated using Equation (10) [41]:

(10) $$Ex_{fan}=P_{fan}\times t$$

The exergy produced by natural gas heating air can be determined by the following Equation (11):

(11) $$Ex_{gas}=m_{g,a}c_{p,a}\left[\left(T_{1}-T_{0}\right)-T_{0}\ln\left(\frac{T_{1}}{T_{0}}\right)\right]$$

To calculate the exergy consumed to dry the material in the drying system can, in turn, be determined using the following formulation (12):

(12) $$Ex_{sys,out}=m_{g,a}c_{p,a}\left(T_{2}-T_{0}\right)-T_{0}\ln\left(\frac{T_{2}}{T_{0}}\right)$$

In the present work, the overall exergy efficiency (*η_ex_*), exergy destruction ratio rate (*R_i,D_*) and exergetic sustainability index (*SI*) were adopted to evaluate the exergy performance of the drying system, and the indices were determined as follows [42,43]:

(13) $$\eta_{ex}=\frac{Ex_{sys,out}}{Ex_{sys,in}}$$

(14) $$R_{i,D}=\frac{Ex_{i,D}}{Ex_{D,total}}$$

(15) $$SI=\frac{1}{\left(1-\eta_{ex}\right)}$$

where *η_ex_* is the ratio of the outlet exergy flow rate to the inlet exergy flow rate of the drying system; *R_i,D_* is the exergy destruction rate of the *i*-th component to the total destruction rate of the whole system and SI is inversely proportional to exergy efficiency.

### 2.8. Exergoeconomic Analysis

**Figure 3:**
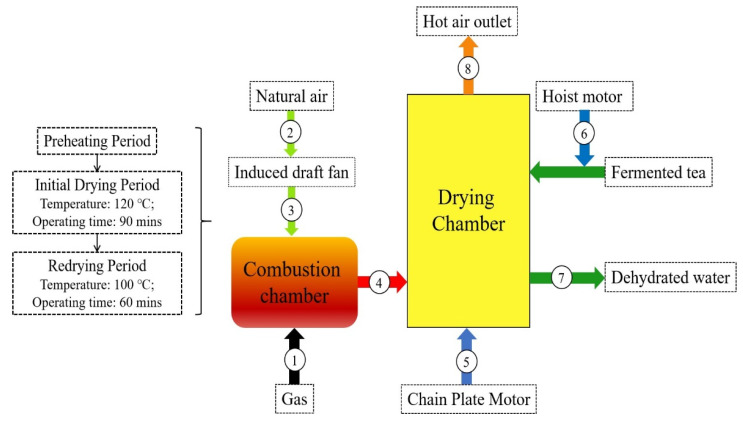
Productive structure of the convective drying system.

The control of the production economic cost is an important index that must be considered in industrial manufacturing. One of the objectives of exergoeconomic analysis is to determine the relationship that includes energy and economic components of the drying system simultaneously, to calculate the associated costs of the inefficiencies of the system and play a guiding role in the selection and design of the components of drying system. Accordingly, as a basic concept of exergoeconomic analysis, the ultimate goal of the exergoeconomic analysis is to convert the economic cost under the action of various exergies into the overall cost of water removal. In the present study, the developed productive structure of the drying system is shown in Figure 3, and the exergy balance equations for each component are shown in Table 4.

Exergoeconomic analysis can be divided into two streams, including the energy–exergy stream and economic stream, respectively, and it is conducted at a component level to calculate the specific costs associated with all exergy streams in the system. Therefore, exergoeconomic can be define as “product cost = energetic cost + nonenergetic cost”; meanwhile, the exergoeconomic analysis can provide important information, which is not easily available from conventional thermodynamic analysis and simple economic evaluations. In terms of the above definition of exergy cost, based on the steady state of each control volume *i* for the total system, a cost balance equation to calculate the exergoeconomic cost for a system can be expressed as the following Equation [44,45,46]:

(16) $$\underset{in}{\sum }C_{i}+Z_{i}^{T}=\underset{out}{\sum }C_{i}+C_{i}^{K}+C_{i}^{Q}$$

where *C_i_* in Equation (16) is the exergoeconomic cost of each stream, which can be determined as Equation (17) [47]:

(17) $$C_{i}=c_{i}Ex_{i}$$

where *c_i_* is the unit exergoeconomic cost of the *i-th* volume.

In the present work, MOPSA, the modified productive structure analysis method, was adopted to calculate the cost of each stream and analyze the exergoeconomic function of the drying system [48]. Based on the analysis presented in Figure 3, the total drying system cost balance equation can be written as:

(18) $$c_{7}Ex_{7}=c_{1}Ex_{1}+\underset{i\in y_{n}}{\sum }Z_{i}$$

This is difference from the traditional method used to analyze the exergoeconomic performance of a drying system; the advantage of adopting the MOSPA method is that it allocates the cost of unusable low-level energy to the corresponding cost of each component in the system. Stream 8 is the cost flow rate of waste from the present system; therefore, the auxiliary equation at the boundary of the overall system can be written as [49]:

(19) $$c_{8}Ex_{8}-c_{r}\underset{i\in y_{n}}{\sum }Ex_{D,i}=0$$

In the present work, *Z_i_* is the energy of the nonenergetic cost, including the investment cost *Z_ic_*, tax cost *Z_tc_* and equipment maintenance cost *Z_mc_*. The corresponding hourly nonenergetic cost *Z_i_* can be calculated as the following Formulas (20)–(22) and the results are tabulated in Table 5 [50]:

(20) $$\dot{Z_{ic}}=\frac{Z_{ic}}{9\times 20\times 40}$$

(21) $$\dot{Z_{tc}}=\frac{Z_{ic}\times 0.1}{9\times 20\times 40}$$

(22) $$\dot{Z_{mc}}=\frac{Z_{ic}\times 0.2}{9\times 20\times 40}$$

According to the energy–exergy and exergoeconomic analyses presented above, the exergoeconomic balanced formulation of each component of the drying system can be expressed as shown in Table 6, and the cost structure matrix formula of the system is calculated using Equation (25). In addition, for the *i-th* component, the exergoeconomic factor *f_c_* and relative cost variance factor *r_c_* were adopted to verify the performance of exergoeconomics and evaluate the energy of the component. The exergoeconomic factor *f_c_*, relative cost variance factor *r_c_* and cost structure matrix formula of the system are calculated using the follow equation [51,52,53]:

(23) $$r_{c,i}=\frac{c_{p,i}-c_{f,i}}{c_{f,i}}$$

(24) $$f_{c,i}=\frac{\dot{Z_{i}}}{\dot{Z_{i}}+c_{f}Ex_{D,i}}$$

(25) $$\left[\begin{array}{ccccc} Ex_{fan}-Ex_{x3} & 0 & 0 & 0 & -Ex_{D,fan}\\ Ex_{3} & -Ex_{4} & 0 & 0 & -Ex_{D,CC}\\ 0 & Ex_{4}-Ex_{8} & Ex_{5}+Ex_{x6} & -Ex_{7} & -Ex_{D,DC}\\ 0 & 0 & Ex_{cpm}-Ex_{5} & 0 & -Ex_{D,cpm}\\ 0 & 0 & Ex_{hm}-Ex_{6} & 0 & -Ex_{D,hm}\\ 0 & Ex_{8} & 0 & 0 & -Ex_{D,\;\mathrm{boundary}\;} \end{array}\right]\times \left[\begin{array}{c} c_{3}\\ c_{4}\\ c_{5}\\ c_{7}\\ c_{r} \end{array}\right]\;=\left[\begin{array}{c} \begin{array}{c} -Z_{fan}\\ -Z_{CC}-c_{1}Ex_{1} \end{array}\\ -Z_{DC}\\ -Z_{cpm}\\ -Z_{hm}\\ 0 \end{array}\right]\;$$

## 3. Results and Discussions

### 3.1. Analysis of Drying Kinetics

According to the description of the structure of the drying equipment presented above, the present experiment adopted the method of a stratified fixed point for the sampling. In the sampling process, the point was set in the middle of each chain plate. Furthermore, we sampled, weighed and recorded the data, after drying and weighing the samples. The data of the samples were used to analyze the drying kinetics, which were normalized to obtain the moisture content data for each layer at a fixed point and are listed in Table 7.

**Figure 4:**
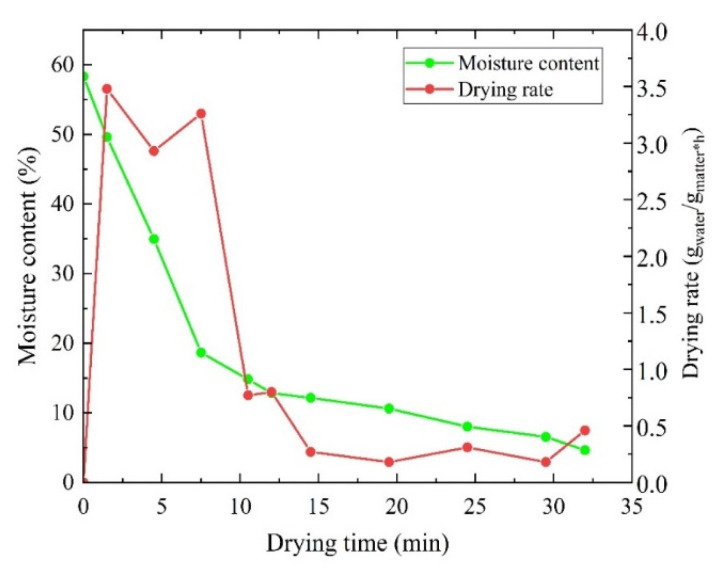
Drying kinetics of the black tea industrial drying process.

The drying kinetics of black tea in an industrial dryer with a drying capacity of 100 kg/h were investigated. In the present work, the drying period from the imported fermented tea to exported dry tea was 32 min, and the moisture content of black tea samples was calculated using the 105 °C constant weight methodology. Figure 4 shows the variations in the moisture content and drying rate with the drying times. It can be observed in Figure 4 that the MC and DR are inversely proportional to the time; the MC of tea in the dryer decreases from a 58.33% wet basis to 4.63% in 32 min. The DR of tea varies from the maximum 3.48 gwater/gdry matter h to a minimum of 0.18 gwater/gdry matter h. In the first 10 min of the drying process, the water removal rate of the entire drying system maintained a high rate, and then decreased and became stable, which might be because the material heated up rapidly at the initial stage of drying, then accelerated the evaporation of the water. In response to this situation, variable-temperature drying technology can be adopted to increase the air temperature in the higher water content drying stage, and reasonably match the supply of energy to improve energy utilization.

### 3.2. Analysis of the Exergetic Performance

In order to identify the performance of components of the black tea drying system and further determine the component optimization of the drying system, the exergetic performance of the components and the whole drying system were investigated. In the present work, the exergy flow rate (*Ex_in_*, *Ex_out_*), exergy destruction rate (*Ex_D_*), exergy efficiency ($\eta_{ex}$), exergetic sustainability index (*SI*) and exergy dissipation ratio (*R_D_*) were adopted to evaluate the exergetic performance of the components and the whole drying system; the results of the above factors are shown in Table 8.

The drying process was divided into the initial drying section and redrying section, the performance index mentioned above was also divided into two sections: the calculation and analysis. As can be observed from Table 9, the exergy efficiency values of the CC and DC were 81.96% and 19.87% for the initial drying section, and 77.69% and 30.33% for the redrying section, respectively. It can be observed that the exergy efficiencies of the CC for the two drying sections were all higher than 75%, indicating that the combustion chamber had a higher efficiency of natural air heating. However, the exergy efficiency of the DC for the two drying sections was lower than 32%, especially the value of 19.87% for the initial drying section. The reason for the lower efficiency value for the initial drying section may be because the exergy loss was relatively high, which was caused by the uneven temperature distribution after the material entered the drying system. On the other hand, the value of the sustainability indices of the CC and DC were 5.54 and 1.25 for the initial drying section, and 4.48 and 1.44 for the redrying section. Furthermore, the exergy dissipation ratios of the CC and DC were 22.11% and 77.89% for the initial drying section, and 28.98% and 71.02% for the redrying section. For the two drying sections, the CC had a higher *SI* than the DC, and the opposite was true for *R_D_*. In addition, the results for the IDF, CPM and HS had exergy efficiency values of 100%, because their energy input to the drying system was electrical energy (the energy–mass coefficient is 1). Therefore, the IDF, CPM and HS can be ignored or ranked last, when considering the rank of improvement priority for the components of the drying system. In general, according to the analysis of the exergy performance of the main energy-consuming components of the drying system, the order of the exergy dissipation ratio of each component from low to high was: IDF, CPM, HS, CC and DC; therefore, the DC should firstly be improved, followed by the CC, HS, CPM and IDF.

**Figure 5:**
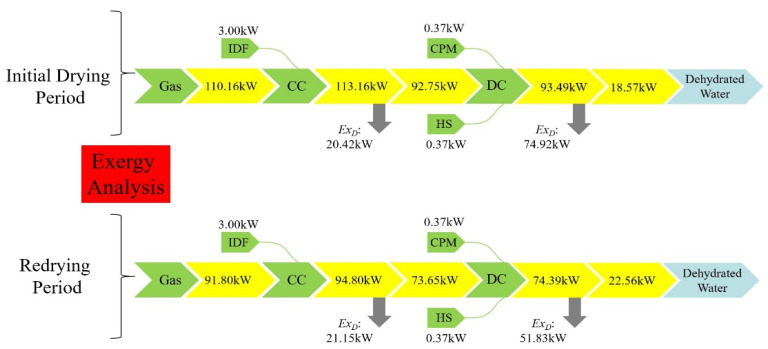
Sankey diagram for the exergy analysis of the overall drying system.

The Sankey diagram for the exergy analysis of the overall drying system is shown in Figure 5, which can visually present the exergy flow direction among the four main components of the entire drying system. As a result of the whole drying operation being divided into two sections, the exergy flow of the drying system can be explained in two parts. As can be observed from Figure 5, in the initial period drying, the initial exergy rate of the CC is 113.16 kW, including the natural air flux, which is considered to be zero. Immediately after this, the exergy output from the CC to DC presented an exergy flow rate value of 93.49 kW. Moreover, there was an obvious exergy destruction rate in the CC, which was 20.42 kW, and 74.92 kW in the DC, respectively, which indicates that the DC can be greatly improved by reducing its exergy destruction rate. Additionally, as described in Figure 5, the situation of the redrying period is the same as the initial drying. As the result of the inlet temperature required for the redrying period was 100 °C, the initial exergy rate in the CC was 94.80 kW and that immediately entering the DC was 74.39 kW. On account of the fact that the temperature of each layer of the DC is relatively stable after the initial drying phase, the exergy destruction values of the CC and DC in the redrying phase were 21.15 kW and 51.83 kW. Compared to the initial drying period, the exergy dissipation rate of the CC and DC were higher than the initial drying period, especially with the difference value of 10.56% in the DC between the initial drying and redrying periods. However, the situation for the initial drying and redrying periods is similar; as a result of this, the exergy efficiency of the DC at any period was the lowest for the whole drying operation, so this should firstly be improved.

### 3.3. Analysis of the Exergoeconomic Performance

In comparison to the previously mentioned ranking analysis of the optimization sequence of the components of the drying system from the perspective of exergy, in this section, the optimization sequence of the components of the drying system can be analyzed from the perspective of exergoeconomics. Therefore, the non-energetic cost *C_i_* and the exergetic cost of the fuel (*c_f,i_*) and the product (*c_p,i_*) for the *i*-th component in the drying system were investigated. At the same time, the relative difference (*r_c,i_*) and the exergoeconomic factor (*f_c,i_*) were also investigated to reveal the relationship between the exergy dissipation rate and investment costs of the components. The results of the indicators mentioned above are provided in Table 9.

Similar to the section in the present study that analyzes the exergetic performances, this section that analyzes the exergoeconomics is also divided into two parts, including the initial drying period and redrying period. Regardless of which period the drying operation was in, the hourly non-energetic costs for each component was consistent, as the result of the investment cost of each component of the drying system was changeless. As shown in Table 9, the sequence of the non-energetic costs for each component of the whole drying system ranked from high to low is the DC, HS, CC, IDF and CPM; their values are 1.08 USD/h, 2.82 × 10^−1^ USD/h, 1.72 × 10^−1^ USD/h, 3.04 × 10^−2^ USD/h and 1.59 × 10^−2^ USD/h, respectively. The non-energetic costs of the DC account for the largest share of the total investment cost, which can be observed in Table 5 that shows that all of the non-energetic costs for the overall system are focused on the DC. It can be observed that *c_f,i_* and *c_p,i_* in the initial drying period are the same in the redrying period, the reason being that the average energy flow of fuel into the furnace is consistent; correspondingly, the result of the *r_c,i_* in the initial drying period is also the same in the redrying period. The exception is that the energy consumed by the IDF, CPM and HS components is electricity; therefore, the value of *c_f,i_* for the IDF, CPM and HS is 0, corresponding to the *r_c,i_* for the IDF, CPM and HS with no value. Among the CC and DC, the highest value of the *r_c,i_* (125.15%) belongs to the DC. The relevant literature indicates that the relative cost difference reveals the potential for discounting the unit cost of the product, and the low exergy efficiency or exorbitant non-energetic costs of the component cause an exorbitant difference in the relative cost. Therefore, in the present study, the unit cost of the DC can be decreased with minimal effort, compared to the CC, which has a lower relative cost difference; the result of the cost difference for the redrying period was the same as the initial drying period. As for the exergoeconomic factors, the DC obtained a higher value of 5.52% than the CC with a value of 3.30% in the initial drying period; in the redrying period, the DC presented a value of 7.78%, compared to the CC that presented a value of 3.19%, except for the special components, such as the IDF, CPM and HS. Table 8 and Table 9 clearly show that *f_c,i_*, for the DC, is influenced by both the component-related cost rate and exergy dissipation; on the other hand, the component-related cost rate is the dominant source for the *f_c,i_* of the DC. Therefore, to improve the exergoeconomics of the whole drying system, efforts should focus on reducing or minimizing the thermodynamic irreversibility of the DC unit and the component-related cost of the CC unit.

**Figure 6:**
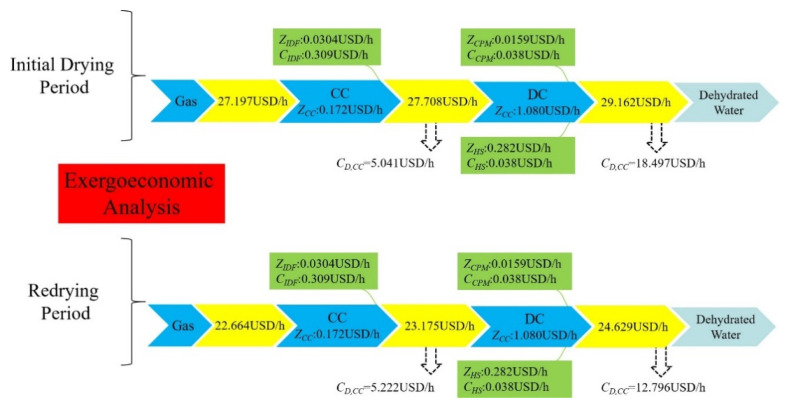
Sankey diagram of the exergoeconomic analysis for the overall drying system.

In the present work, the unit exergoeconomic of the fresh air stream was assumed to be zero; the hourly economic costs of the components of the drying system are calculated and shown in Figure 6. From Figure 6, it can be observed that the hourly economic cost for dehydrating moisture is 29.162 USD/h in the initial drying section, and 24.692 USD/h in the redrying section. As the main operating unit component of the drying system, the component-related cost rate of the DC was not only the major source for the total cost rate, but also the dominant source for the exergy destruction rate. The component-related cost rate of the DC was 1.080 USD/h, and the costs of the exergy destruction rates were 18.497 USD/h for the initial drying section and 12.796 USD/h for the redrying section. Therefore, economic improvement should focus its efforts on reducing the DC component-related costs and exergy destruction costs.

## 4. Conclusions

In the present study, the cost rate of dehydrated water was considered as a final goal to evaluate the economic performance of a drying system. The drying kinetics, and exergetic and exergoeconomic performances of the drying system were discussed. According to the results obtained from the above analysis, the following conclusions can be drawn:

- The drying rate of tea varied from the maximum value of 3.48 g_water_/g_dry matter_ h to the minimum value of 0.18 g_water_/g_dry matter_ h. More specifically, in the first 10 min of the initial drying period, the moisture content of the material was high and the temperature rapidly rose, resulting in the water removal rate to accelerate to obtain the highest drying rate with the value of 3.48 g_water_/g_dry matter_ h in the system.
- In the initial drying system, the exergy destruction mainly occurred in the CC and DC, the values of the exergy destruction rates of the CC and DC were 20.42 kW and 74.92 kW and the exergy efficiency values were 81.96% and 19.87%; whereas, in the redrying period, the values of the exergy destruction rates were 21.15 kW and 51.83 kW, and the exergy efficiency values were 77.69% and 30.33%.
- From the perspective of exergoeconomics: The DC had the highest values for the cost of the exergy destruction rate (18.497 USD/h) for the initial drying period and 12.796 USD/h for the redrying period; followed by the CC with the values of 5.041 USD/h for the initial drying period and 5.222 USD/h for the redrying period.
- The DC was determined to have the highest exergoeconomic importance based on the total capital investment and exergy destruction cost rate. A comprehensive analysis of the exergy and exergoeconomics factors was conducted, in order to obtain a cost-effective system; the order of the optimal performance of each component of the drying system ranked from low to high was IDF, CPM, HS, CC and DC; therefore, the DC is considered to improve firstly, followed by the CC, HS, CPM and IDF.
- The unit exergy rate consumed by the drying system to remove water was determined with the value of 18.57 kW in the initial drying period and 22.56 kW in the redrying period; in addition, the values of the unit drying cost of the drying system were determined as 29.162 USD/h in the initial drying section and 24.629 USD/h in the redrying section.

In this study, the exergy and economic performances of the existing gas-type industrial drying system of black tea were analyzed. Therefore, the results obtained in the present work can not only help the staff to evaluate and optimize the drying system from the perspective of energy and economy, but can also identify the components that can reduce or minimize the cost and economic flow rates. However, there are many deficiencies in the current work. Thus, it is recommended that the distribution changes in the temperature and humidity in the drying warehouse be studied more extensively to further enhance the equipment structure, to improve the energy efficiency and reduce the economic costs. Furthermore, the relationship between the system exergy and the exergoenvironment should also be studied.

## Figures and Tables

**Table 1:** Recent works on energy and exergy analyses for agricultural product drying systems.

Agr. Product	Drying System	Main Conclusions	References
Cassava starch	Tray dryer	Energy efficiency increased from 16.036 to 30.645%, and exergy inflow, outflow and losses increased from 0.399 to 2.686, 0.055 to 0.555 and 0.344 to 2.131 J/s, respectively, in the above temperature range.	[14]
Rough rice	Fluidized bed	The energy and the exergy efficiencies increased with increasing the drying air temperature and solid holdup, whereas they decreased with the increase in superficial fluidization velocity.	[15]
Banana	Indirect-type natural convection solar dryer	The exergy losses varied from 3.36 to 25.21 kJ/kg. In particular, the exergy efficiency values varied from 7.4 to 45.32%.	[16]
Cassava chips	Multipurpose convective tray dryer	The energy utilization, exergy inflow, exergy outflow and exergy efficiency increased in the ranges of 9.53–24.66 kJ/s, 5.67–11.34 kJ/s, 2.21–8.04 kJ/s and 38.90–270.86%.	[17]
Stevia leaves	Forced convection solar dryer	The overall dryer and average exergy efficiencies of the MFSCD were 33.5% and 59.1%.	[18]

**Table 2:** Operation date.

Project	Values
Tea-drying month for a year (month/year)	9
Machine running time (hours/month)	40
Economic life (year)	20
Annual output of dry tea (kg/year)	9000
Local market price of dry tea (USD/kg)	157
Price of natural gas (USD/t)	2523
Electricity price for industrial production (USD/kWh)	0.103
Currency exchange rate: 6.34 RMB = USD 1	

**Table 3:** Details of the experimental instruments.

Instrument	Type	Measurement	Instrument
Thermal resistance	PT100	−200–450 °C	±0.1 °C
Temperature and humidity sensors	AM2301	0–100%/−40–80 °C	±3%/±0.5 °C
Paperless recorder	GTM302C	−250–600 °C	±0.1 °C
Electronic scale	ABJ 320-4NM	0–380 g	±0.01 g
Constant-temperature drying box	DGG-9070A	105 °C	±0.1 °C

**Table 4:** Fuel exergy, product exergy, exergy dissipation and exergy efficiency of the components of the system.

Components	Fuel Exergy	Product Exergy	Exergy Dissipation	Exergy Efficiency
IDF	*Ex_fan_* + *Ex_2_*	*Ex_3_*	*Ex_fan_* + *Ex_2_* − *Ex_3_*	*Ex_3_*/(*Ex_fan_* + *Ex_2_*)
CC	*Ex_1_* + *Ex_3_*	*Ex_4_*	*Ex_1_* + *Ex_3_* − *Ex_4_*	*Ex_4_/*(*Ex_1_* + *Ex_3_*)
DC	*Ex_4_* + *Ex_5_* + *Ex_6_*	*Ex_7_*	*Ex_4_* + *Ex_5_* + *Ex_6_* − *Ex_7_* − *Ex_8_*	*Ex_7_*/(*Ex_4_* + *Ex_5_* + *Ex_6_* − *Ex_8_*)
CPM	*Ex_cpm_*	*Ex_5_*	*Ex_cpm_* − *Ex_5_*	*Ex_5_*/*Ex_cpm_*
HS	*Ex_hm_*	*Ex_6_*	*Ex_hm_* − *Ex_6_*	*Ex_6_*/*Ex_hm_*

Note: The induced draft fan, chain plate motor and the hoisting motor convert the electrical energy into the corresponding energy required by the drying system: *Ex_fan_* = *Ex_2_*, *Ex_cpm_* = *Ex_5_*, *Ex_hm_* = *Ex_6_*.

**Table 5:** Non-energetic costs of the subsystems.

Subsystem	*Z_ic_* (USD)	$\dot{Z_{ic}}(\mathrm{USD}/h)$	$\dot{Z_{tc}}(\mathrm{USD}/h)$	$\dot{Z_{mc}}(\mathrm{USD}/h)$	Total Non-Energy Cost (USD/h)
IDF	195.27	2.71 × 10^−2^	2.71 × 10^−3^	5.42 × 10^−4^	3.04 × 10^−2^
CC	1110.09	1.54 × 10^−1^	1.54 × 10^−2^	3.08 × 10^−3^	1.72 × 10^−1^
DC	6940.06	9.64 × 10^−1^	9.64 × 10^−2^	1.93 × 10^−2^	1.08
CPM	102.52	1.42 × 10^−2^	1.42 × 10^−3^	2.84 × 10^−4^	1.59 × 10^−2^
HS	1813.88	2.52 × 10^−1^	2.52 × 10^−2^	5.04 × 10^−3^	2.82 × 10^−1^
Whole system	10161.82	1.41	1.41 × 10^−1^	2.82 × 10^−2^	1.58
Currency exchange rate: 6.34 RMB = 1 USD					

**Table 6:** Cost balance equations; F rule and arbitrary assumptions computed for all of the components of the drying system.

Components	Cost Balance	Unit Exergoeconomic Cost
IDF	*c_fan_Ex*_fan_ + *c_2_Ex*_2_ − *c_3_Ex*_3_ − *c_r_Ex_D,fan_ + Z_fan_* = 0	*c_fan_* = *c_3_* = 28.61 *USD/GJ*; *c_2_* = 0;
CC	*c_1_Ex*_1_ + *c_3_Ex_3_* − *c_4_Ex*_4_ − *c_r_Ex_D,CC_ + Z_CC_* = 0	*c_1_* = 68.58 *USD/GJ*; *c_4_* = *c_8_*(F-rule)
DC	*c_4_Ex_4_* + *c_5_Ex_5_* + *c_6_Ex_6_* − *c_7_Ex_7_* − *c_8_Ex_8_* − *c_r_Ex_D,DC_ + Z_DC_* = 0	*c_5_* = *c_6_* = 28.61 *USD/GJ*; *c_7_* = (final product exergy cost);
CPM	*c_cpm_Ex_cpm_* − *c_5_Ex_5_* − *c_r_Ex_D,cpm_ + Z_cpm_* = 0	*c_cpm_* = *c_5_*
HS	*c_hm_Ex_hm_* − *c_6_Ex_6_* − *c_r_Ex_D,hm_ + Z_hm_* = 0	*c_hm_* = *c_6_*
Currency exchange rate: 6.34 RMB = 1 USD		

**Table 7:** Mass of the tea sampling in the drying processing.

Drying Section	Time	Layer	Mass
	mins		g
**Initial drying period (120 °C)**	0	Initial	42.69
	1.5	L4	35.31
	4.5	L3	27.35
	7.5	L2	21.87
	10.5	L1	20.88
	12	Initial dried tea	20.41
**Redrying period (100 °C)**	14.5	L4	20.25
	19.5	L3	19.90
	24.5	L2	19.34
	29.5	L1	19.04
	32	Re-dried tea	18.65

**Table 8:** The exergetic performance of the components for the overall drying system.

Drying Section	Components	*Ex_sys,in_* (kW)	*Ex_sys,out_* (kW)	*Ex_D_* (kW)	$\eta_{ex}(\%)$	*SI*	*R_D_* (%)	Improvement Priority
**Initial drying period (120 °C)**	IDF	3	3	0	100	/	0	3
	CC	113.16	92.75	20.42	81.96	5.54	22.11	2
	DC	93.49	18.57	74.92	19.87	1.25	77.89	1
	CPM	0.37	0.37	0	100	/	0	3
	HS	0.37	0.37	0	100	/	0	3
**Redrying period (100 °C)**	IDF	3	3	0	100	/	0	3
	CC	94.80	73.65	21.15	77.69	4.48	28.98	2
	DC	74.39	22.56	51.83	30.33	1.44	71.02	1
	CPM	0.37	0.37	0	100	/	0	3
	HS	0.37	0.37	0	100	/	0	3

**Table 9:** The exergoeconomic performance of the components for the overall drying system.

Drying Section	Components	*Zi* (USD/h)	*c_f,I_* (USD/GJ)	*c_p,I_* (USD/GJ)	*r_c,I_* (%)	*f_c,I_* (%)	Improvement Priority
**Initial drying period (120 °C)**	IDF	3.04 × 10^−2^	0	28.61	/	100	1
	CC	1.72 × 10^−1^	68.58	97.19	41.72	3.30	5
	DC	1.08	68.58	154.41	125.15	5.52	4
	CPM	1.59 × 10^−2^	0	28.61	/	100	1
	HS	2.82 × 10^−1^	0	28.61	/	100	1
**Redrying period (100 °C)**	IDF	3.04 × 10^−2^	0	28.61	/	100	1
	CC	1.72 × 10^−1^	68.58	97.19	41.72	3.19	5
	DC	1.08	68.58	154.41	125.15	7.78	4
	CPM	1.59 × 10^−2^	0	28.61	/	100	1
	HS	2.82 × 10^−1^	0	28.61	/	100	1

## Data Availability

Not applicable.

## Nomenclature

*MC_wb_*	Moisture-content wet basis (%)
*DR*	Drying rate (g_water_/g_matter*h_)
*m_wet_*	Mass of wet material (g)
*m_d_*	Mass of dry material (g)
*m_g,a_*	Mass flow rate of air (kg·s^−1^)
*t*	Time (min)
*Q_gas_*	Heat of gas (J)
*V_gas_*	Unit volume of natural gas (m^3^)
*q_gas_*	Calorific value of gas (J/m^3^)
*m_g,a_*	Mass flow of air (kg/s)
*c_p,a_*	Specific heat of air (J kg^−1^ °C^−1^)
*Ex_sys,in_*	Inlet exergy of drying system (kW)
*Ex_sys,out_*	Outlet exergy of drying system (kW)
*Ex_sys,des_*	Exergy dissipation of drying system (kW)
*Ex_gas_*	Exergy of gas (kW)
*Ex_fan_*	Exergy of fan (kW)
*η_ex_*	Exergy efficiency
*R_i,D_*	Exergy destruction ratio
*SI*	Exergetic sustainability index
*T_1_*	Temperature of inlet ( °C)
*T_2_*	Temperature of outlet ( °C)
*T_0_*	Temperature of ambient ( °C)
*Q_a_*	Air heating (J)
*Q_loss_*	Heated air heat loss (J)
*P_fan_*	Power of fan (kW)
*W_fan_*	Work of fan (kJ)
CC	Combustion chamber
IDF	Induced draft fan
DC	Drying chamber
HS	Hoist motor
CPM	Chain plate motor
PP	Preheating period
IDP	Initial drying period
RP	Redrying period
